# 1882. Effects of BA.1/BA.2 Subvariant, Vaccination, and Prior Infection on Infectiousness of SARS-CoV-2 Omicron Infections

**DOI:** 10.1093/ofid/ofac492.1509

**Published:** 2022-12-15

**Authors:** Suelen Qassim, Laith J Abu-Raddad, Hiam Chemaitelly, Sawsan AlMukdad

**Affiliations:** Weill Cornell Medicine-Qatar, Doha, Ad Dawhah, Qatar; Weill Cornell Medicine, Doha, Ad Dawhah, Qatar; Weill Cornell Medicine - Qatar, Doha, Ad Dawhah, Qatar; Weill Cornell Medicine-Qatar, Doha, Ad Dawhah, Qatar

## Abstract

**Background:**

Qatar experienced a large SARS-CoV-2 Omicron (B.1.1.529) wave that started on December 19, 2021 and peaked in mid-January, 2022. We investigated effects of Omicron subvariant (BA.1 and BA.2), previous vaccination, and prior infection on infectiousness of Omicron infections, between December 23, 2021 and February 20, 2022. The RT-qPCR cycle threshold (Ct) value of a SARS-CoV-2 infection represents the inverse of viral load and is correlated with culturable virus; thus, it can be used as a proxy for SARS-CoV-2 infectiousness.

**Methods:**

The study population included all individuals with an RT-qPCR-confirmed SARS-CoV-2 infection in Qatar in the study period. All relevant data for this population were extracted from the national, federated SARS-CoV-2 databases. A SARS-CoV-2 infection with BA.1 was proxied as an S-gene “target failure” (SGTF) case, whereas infection with BA.2 was proxied as a non-SGTF case. The average Ct values of the N, ORF1ab, and S (if not an SGTF case) genes was used as the dependent variable. Univariable and multivariable regression analyses were conducted to estimate the association between Ct value and each of the Omicron subvariants, mRNA vaccination, prior infection, reason for RT-qPCR testing, study-period week of RT-qPCR test, and demographic factors.

**Results:**

Compared to BA.1, BA.2 was associated with 3.53 fewer cycles (95% CI: 3.46-3.60), signifying higher infectiousness (Table 1). Ct value decreased with time since second and third dose vaccinations. Ct values were highest for those who received their boosters in the month preceding the RT-qPCR test—0.86 cycles (95% CI: 0.72-1.00) higher than for unvaccinated persons. Ct value was 1.30 (95% CI: 1.20-1.39) cycles higher for those with a prior infection compared to those without prior infection, signifying lower infectiousness.

**Figure:**
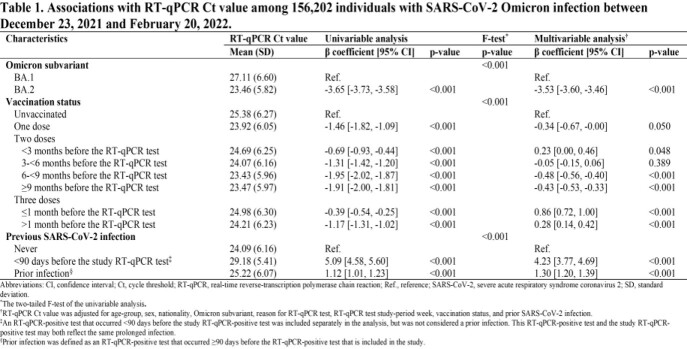

**Conclusion:**

BA.2 appears to be associated with substantially higher infectiousness than BA.1. This may reflect higher viral load and/or longer duration of infection, thereby explaining the rapid expansion of this subvariant in Qatar. Natural immunity from previous infection and strength of vaccine immunity correlated with less infectious breakthrough infections.

**Disclosures:**

**All Authors**: No reported disclosures.

